# Bridging cultural divides: family support and psychological resilience as catalysts for student adaptability in Sino-foreign cooperative education

**DOI:** 10.3389/fpsyg.2025.1717876

**Published:** 2025-12-04

**Authors:** Yiwen Yan, Moudi Mahdi, Yuan He

**Affiliations:** 1 School of Management, Chengdu University of Information Technology, Chengdu, China; 2 College of Culinary and Food Science Engineering, Sichuan Tourism University, Chengdu, China; 3 College of Resources and Environment, Chengdu University of Information Technology, Chengdu, China

**Keywords:** Sino-foreign cooperative education, family support, cross-cultural learning, adaptability, psychological resilience

## Abstract

**Introduction:**

With the rapid advancement of educational internationalisation, Sino-foreign cooperative education has become a vital mechanism for reform, though students’ insufficient cross-cultural adaptability poses a significant challenge to their academic excellence and international skill acquisition.

**Methods:**

This study randomly selected and surveyed 402 students from eight Sino-foreign cooperative educational projects in Chengdu, utilising structural equation modelling to investigate the factors affecting student adaptability.

**Results:**

The findings reveal that family support has a significantly positive impact on students’ cross-cultural adaptability, with psychological resilience acting as a key intermediate variable in this relationship. This underscores the critical combination of external aids and internal psychological assets.

**Discussion:**

Consequently, it is suggested that a family-based support system be established, and psychological education alongside cross-cultural training be enhanced to bolster students’ psychological resilience and adaptive capacity. Furthermore, the management of these joint programs should be improved to cultivate international talents who are well-adapted to the demands of socio-economic advancement.

## 1 Introduction

In recent years, as educational internationalization has progressed, Sino-foreign cooperative education has emerged as a critical component of higher education’s international development in China. The history of Sino-foreign cooperative education can be traced back to the early 1990s. Following the continuous expansion of China’s open-door policy, international educational cooperation has become a crucial instrument for elevating higher education standards. Entering the 21st century, the government enacted a series of policies to foster the development of Sino-foreign cooperative education. In 2003, the “Regulations on Sino-Foreign Cooperative Education” established a legal framework and policy support, catalyzing a surge in cooperative projects and diversification of educational models. Numerous domestic universities have actively partnered with overseas institutions to develop diverse educational programs, importing high-quality international educational resources to enhance both educational quality and institutional competitiveness. With the enactment of the national strategy for the development of western regions and the “Belt and Road” initiative, Chengdu has progressively emerged as a central hub for Sino-foreign cooperative education in Western China. As of July 2024, the Ministry of Education’s Sino-Foreign Cooperative Education Supervision Platform reports that Sichuan Province hosts seven cooperative education institutions and 28 cooperative education projects, including one that encompasses the Hong Kong, Macau, and Taiwan regions. Chengdu, serving as the economic and cultural hub of Southwest China, has attracted numerous Sino-foreign cooperative educational projects. Chengdu plays a pivotal role in these initiatives, facilitating programs at various educational levels from undergraduate to postgraduate across multiple disciplines such as civil engineering, computer science and technology, and film and television production. Chengdu has accumulated substantial experience in Sino-foreign cooperative education. Notably, the collaboration between Southwest Jiaotong University and Leeds College encompasses five majors—civil engineering, mechanical design and manufacturing automation, electronic information engineering, computer science and technology, and materials science and engineering. These programs have received accreditation from the Chinese Engineering Education Accreditation Network, aiming to foster internationally competitive, high-quality innovative engineering talents. In 2024, this collaboration was designated as a demonstration unit for Sino-foreign cooperative institutions. Furthermore, Chengdu University’s Stirling College offers three undergraduate majors in leisure sports, data science and big data technology, and network and new media. These programs are committed to developing internationalized, high-quality applied talents who are urgently needed in the health and sports industry, well-versed in international standards, and equipped with both national pride and a global perspective. However, amid cultural integration, the adaptability of students has proven essential in influencing their academic outcomes as well as their physical and mental wellbeing. Research indicates a strong correlation between students’ adaptability and factors such as psychological traits and family support (Wilks and Spivey, 2010; Turner et al., 2022). Adaptability not only impacts academic performance but may also exert a long-term effect on career development. Consequently, examining the factors that influence student adaptability holds significant theoretical and practical relevance for enhancing the quality of Sino-foreign cooperative education programs.

Although extensive research has been conducted on the relationship between student adaptability and factors such as family support and psychological resilience, these studies have predominantly focused on students in traditional educational settings. Relatively few investigations have examined students in Sino-foreign cooperative education programs. The existing literature primarily addresses three areas: firstly, the exploration of the basic concepts and determinants of student adaptability, offering an in-depth analysis of the development and formation of this adaptability (Argyros, 2019; García-Martínez et al., 2022); secondly, the direct impact of family support on students’ psychological wellbeing and behavior (Parker et al., 2021); and thirdly, the role of psychological resilience as a personal resource in managing stress and challenges (Shi et al., 2018; Ke et al., 2022). While these studies provide valuable insights into student adaptability, the specific mechanisms through which family support and psychological resilience influence adaptability within the context of Sino-foreign cooperative education programs remain to be elucidated.

Even if the success achieved in the Sino-foreign cooperative education system in Chengdu is quite remarkable, the aspect of cultural difference management and education quality has been a concern. For this reason, the aim of this particular piece of research will endeavor to comprehend the impact of psychological resilience and family support towards the adaptability of Sino-foreign cooperative education students. In particular, the aim of the current research will focus greatly on Sino-foreign cooperative education program students based in Chengdu. The main objective of the current piece of research will be to illuminate the link between family support and the adaptability of students.

## 2 Theoretical framework and research hypotheses

### 2.1 Analysis of the mechanisms affecting student adaptability

Adaptability is an individual’s capacity to manage stress, challenges, or difficult situations and serves as a key indicator of mental health and quality of life. Research indicates that adaptability is shaped by a combination of internal and external factors (Han and Zhang, 2023). External factors primarily include social support, life events, and the family environment. Internal factors consist of psychological resilience and self-esteem (Semkovska et al., 2022; Tang et al., 2022). Notably, family support plays a crucial external role in influencing adaptability. Positive family dynamics can alleviate stress, fulfill emotional needs, and assist individuals in more confidently and effectively confronting academic and life challenges. Psychological resilience, a significant internal factor, enables individuals with high resilience to adapt effectively in the face of adversity.

**Figure 1:**
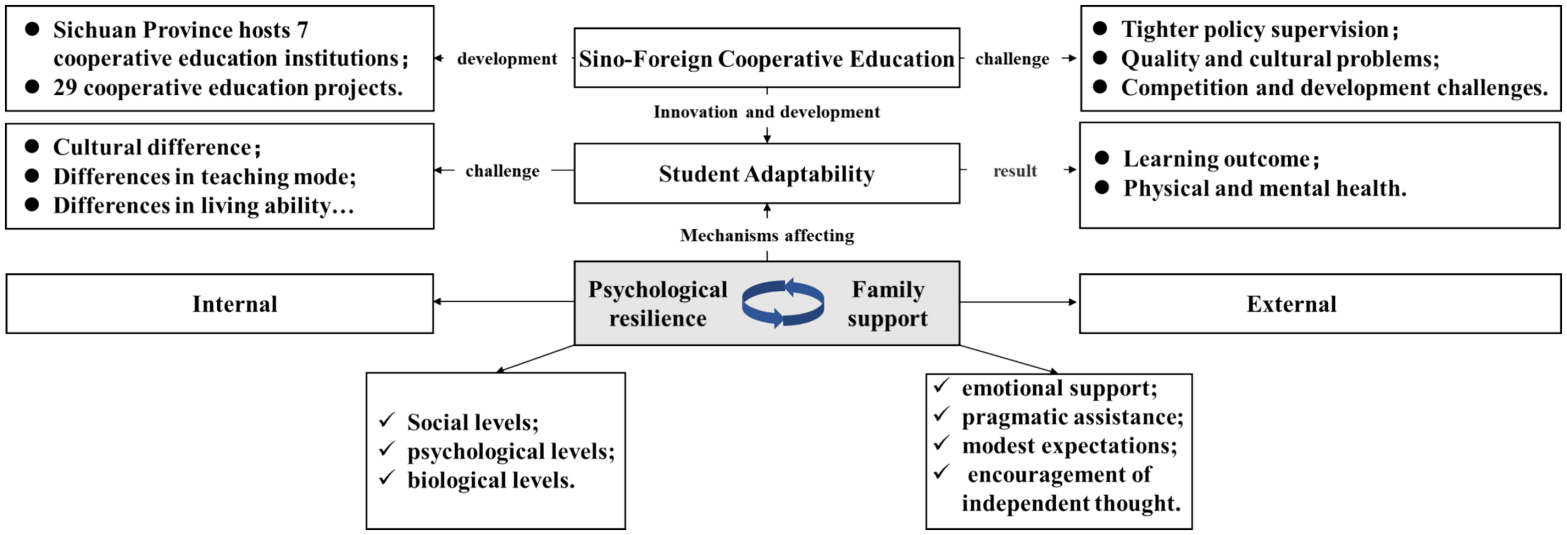
Theoretical framework diagram.

As illustrated in Figure 1, the theoretical model integrates these internal and external factors. The model illustrates how family support and psychological resilience are connected and interrelated to collectively determine student adaptability. The diagram illustrates how these factors operate within the overall context of Sino-foreign cooperative education with an emphasis on the interrelation between family support and psychological resilience in determining students’ adaptability.

Family support, as a pivotal form of social support, significantly influences students’ mental health and adaptability. Dina and Putra (2022) found that family support profoundly affects students’ career decision-making, suggesting that students with strong family support are more confident in making career choices. Wang et al. (2024) further identified that career decision-making self-efficacy serves as a mediating factor between family support and students’ career adaptability, underscoring the critical role of family support in enhancing students’ confidence and adaptability in their careers. In their 2022 study, Martins and Moriña (2023) examined students with disabilities and concluded that family support is crucial in helping these students overcome life challenges and improve their adaptability, especially in higher education settings. Fitriani and Gina (2022) researched the impact of family support on students’ academic resilience and adaptability post-pandemic, finding that family support significantly enhances students’ coping skills and academic adaptability. Salwani and Cahyawulan (2022) highlighted the positive effect of family support on students’ self-confidence, further demonstrating the importance of family in students’ career development.

In recent years, psychological resilience has emerged as a key focus in research exploring factors influencing adaptability. Extensive studies have examined the interplay between individual adaptability and psychological resilience. Denckla et al. (2020) highlighted that the concept of psychological resilience has evolved; it is no longer seen merely as a set of individual traits but also as involving dynamic interactions across various systems, impacting students’ adaptability on social, psychological, and biological levels. Franco Buitrago et al. (2023) conducted research at a Colombian university with students of differing abilities and found that psychological resilience significantly aids students in adapting to campus life, particularly when facing academic and social challenges. Najafi and Ebrahimi Belil (2023) suggested that social support and self-esteem directly enhance students’ psychological resilience, thereby improving their adaptability in managing chronic family illnesses. Mahmud et al. (2022) also discovered that family support positively influences students’ career adaptability, underscoring the strong linkage between family support and adaptability in higher education. Hirano (2020) observed that resilience varies among individuals confronted with identical stressors, influenced not only by internal traits but also by external factors such as culture and environment. Supporting this notion, Ungar et al. (2021) introduced the concept of multi-system resilience, emphasizing that psychological resilience, when integrated with other factors like environment and social support, effectively enhances individual adaptability. These findings reveal a complex interrelation between family support and psychological resilience, collectively shaping students’ adaptability. It is evident that family support can facilitate adaptability through psychological resilience, and in turn, psychological resilience is shaped by family support.

Based on these insights, the following hypotheses are proposed:

*H1*: There is a significant correlation between family support and student adaptability.

*H2*: Psychological resilience is significantly correlated with student adaptability.

*H3*: Psychological resilience fully mediates the effect of family support on student adaptability.

**Figure 2:**
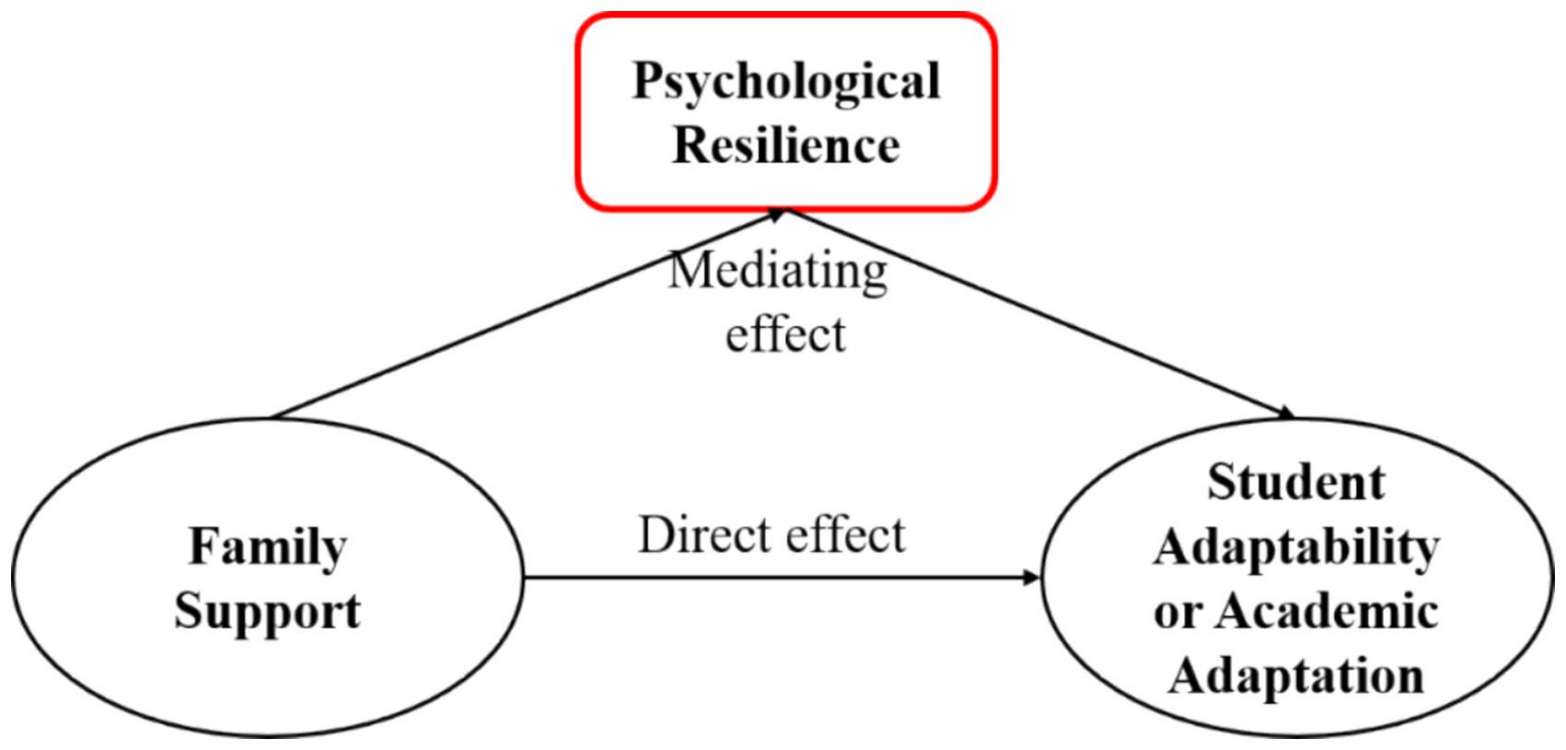
Relationship diagram of intermediary role.

Based on the aforementioned assumptions, this article constructs a diagram illustrating the relationship among family support, psychological resilience, and student adaptability, as depicted in Figure 2.

## 3 Research methods

### 3.1 Participants

This study employed cluster sampling to select 450 undergraduate students from eight Sino-foreign cooperative education programs in Chengdu, Sichuan Province, as survey participants. A total of 450 questionnaires were distributed, of which 440 were returned, resulting in 402 valid responses, yielding a response rate of 91.4%. The mean age of the sample was 20.67 ± 2.81 years. Table 1 represents the respondents’ demographic profile.

**Table 1:** Respondents’ profile.

Category		Frequency (402)	Percentage (%)
Gender	Male	174	43.3
	Female	228	56.7
Grade	First year	129	32.1
	Sophomore year	124	30.8
	Junior year	104	25.9
	Senior class	45	11.2

### 3.2 Measures

#### 3.2.1 Student adaptability scale

The above scale was designed for the measurement of the adaptability of students within a cross-cultural learning setting and assesses their performance in the context of culture adaptation, social adaptation, and emotional adaptation. The adapted scale contains 15 questions and three aspects, namely culture adaptation, social adaptation, and emotional adaptation. A five-point scaling technique was used where “1” indicates “strongly disagree” and “5” indicates “strongly agree for the response.” In the proposed study, the alpha value for the total adapted scale stands at 0.742.

#### 3.2.2 Family support scale

The scale is majorly designed to estimate the level of support the students might be getting from their family background based on the emotional, tangible, and interaction support from the family. The scale comprises a total of 12 items, which are grouped based on three themes, including emotional support, tangible support, and interaction support. A five-point scaled score from the Likert scale method is used, where the score “1” represents “strongly disagree” and the score “5” represents “strongly agree.” In the study, the total *α* level for the scaled score comes to be approximately 0.732.

#### 3.2.3 Psychological resilience scale

This scale was used for measuring the psychological resilience of students against challenges and stresses, for the assessment of toughness, positive cognition, and emotional regulation skills of the students. The scale comprises a total of 24 items, distributed evenly over the three dimensions of toughness, positive cognition, and emotional regulation. A high score reveals greater psychological resilience among students enrolled in Sino-foreign cooperative education programs. A five-point scaled score from the Likert scale method is used, where the score “1” represents “strongly disagree” and the score “5” represents “strongly agree.” The total Cronbach’s alpha value for the scale in the proposed study equals 0.826.

### 3.3 Data processing

Firstly, the gathered information from the conducted survey was processed in the SPSS version 24.0 software. The aim of the software here was the generation of descriptive statistics focusing on the measurement of the central tendency and dispersion of the variables used in the study. Descriptive statistics were employed in the computation of the mean (M) and the standard deviations (SD) of each of the variables used. Secondly, the Pearson correlation test was employed in determining the relationship between adaptability among the students, the psychological resilience of the students, and the support the students get from their families. The outcome of the correlation test revealed the correlation between the two factors as inter-dependent. Thirdly, the AMOS software version 24.0 rated *p* < 0.05.

## 4 Results analysis

### 4.1 Descriptive statistics

As illustrated in Table 2, significant correlations exist between family support, psychological resilience, and student adaptability. Emotional support, tangible support, and interactive support each positively correlate with various dimensions of psychological resilience. Notably, emotional support exhibits the strongest influence on emotional control (*r* = 0.22), suggesting that familial emotional support enhances the emotional regulation abilities of students in Sino-foreign cooperative education programs. Moreover, each facet of family support significantly affects the three aspects of student adaptability, with emotional support showing the most pronounced impact on emotional adaptation (*r* = 0.32). The components of psychological resilience, tenacity, positive cognition, and emotional control, also display positive correlations with different dimensions of student adaptability. The strongest correlation observed is between emotional control and emotional adaptation (*r* = 0.45), indicating that robust emotional control skills are essential for students’ emotional adaptation. Consequently, Hypotheses H1 and H2 are supported.

**Table 2:** Results of sample statistics and correlation analysis.

Variables	M ± SD	1	2	3	4	5	6	7	8	9
1. Emotional support	3.85 ± 0.72	1								
2. Tangible support	3.68 ± 0.76	0.30**	1							
3. Interactive support	3.55 ± 0.78	0.25**	0.35**	1						
4. Resilience	4.05 ± 0.65	0.20*	0.28**	0.22*	1					
5. Positive cognition	3.88 ± 0.70	0.18*	0.24**	0.26**	0.43**	1				
6. Emotional regulation	3.92 ± 0.69	0.22*	0.19*	0.21*	0.47**	0.42**	1			
7. Cultural adaptation	3.80 ± 0.74	0.28**	0.23*	0.19*	0.38**	0.31**	0.34**	1		
8. Social adaptation	3.90 ± 0.71	0.26**	0.21*	0.24**	0.40**	0.35**	0.36**	0.42**	1	
9. Emotional adaptation	3.85 ± 0.73	0.32**	0.30**	0.28**	0.44**	0.39**	0.45**	0.40**	0.43**	1

** and * represent statistical significance at the 5% and 10% levels, respectively.

In summary, family support significantly influences both students’ psychological resilience and their direct adaptability. Psychological resilience serves as a crucial internal resource, enabling students to effectively manage environmental changes. Consequently, enhancing family support and psychological resilience represents a strategic approach to improving students’ adaptability.

### 4.2 The impact of psychological resilience on student adaptability: mediation effect analysis

**Figure 3:**
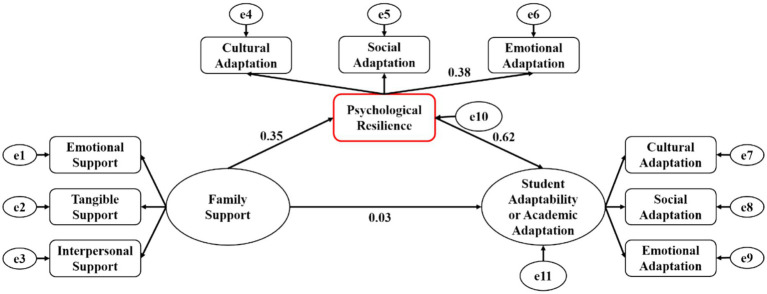
Path coefficients of the mediation model.

Correlation analysis from Table 2 reveals significant relationships among family support, psychological resilience, and student adaptability within the context of Chengdu’s Sino-foreign cooperative education programs. However, the specific processes and predictive effects of these relationships warrant further exploration. This study introduces Hypothesis H3, positing that psychological resilience fully mediates the relationship between family support and student adaptability. To substantiate this hypothesis, a structural equation model is employed to rigorously analyze the interrelations among family support, psychological resilience, and student adaptability, drawing on the aforementioned correlation analysis. This model was developed using Amos 24.0, and path analysis was performed to scrutinize its functional mechanisms. The model presented in this study is depicted in Figure 3, with the fit indices for the model displayed in Table 3. The results presented in Table 3 indicate that the model developed in this study exhibits satisfactory fit indices and meets the established criteria for acceptance.

**Table 3:** Fitting indices of the mediation model for psychological resilience.

*χ* ^2^	Df	*χ*^2^/df	RMSEA	GFI	AGFI	NFI	CFI
61.905	22	2.814	0.067	0.969	0.937	0.913	0.941

For students in Chengdu’s Sino-foreign cooperative education programs, the direct predictive impact of family support on student adaptability is 0.03 (*p* > 0.05), suggesting it is not statistically significant. For the estimation of the indirect effect, the Bias-Corrected Bootstrap method was employed with 5,000 resamples. The indirect predictive effect of family support on adaptability of students through resilience was 0.38. This explains 92.7% of the total effect. The confidence interval for the indirect effect was [0.27, 0.45], and the interval does not contain zero. This indicates the significance at the 95% confidence level. This demonstrates that psychological resilience serves as a full mediator in the relationship between family support and student adaptability. In essence, the direct influence of family support on the adaptability of students in these programs is relatively minimal; its primary effect is mediated through psychological resilience. Therefore, Hypothesis H3 is supported.

## 5 Discussion

### 5.1 The relationship between family support and student adaptability

This study reveals that family support significantly impacts the adaptability of students in Sino-foreign cooperative education programs. Emotional support, tangible support, and interactive support each play a significant positive role in various aspects of student adaptability. Notably, emotional support is crucial for students’ emotional adaptation, which is consistent with the findings of Wanqing et al. (2022). Their research indicates that family emotional support enables students to better manage their emotions and adapt to cross-cultural learning challenges (Wanqing et al., 2022). Similarly, Zheng and Zhou’s (2022) study corroborates these findings, demonstrating that emotional support positively influences students’ emotional regulation and learning status in Sino-foreign cooperative education settings. Such discoveries form the basis for the most important positive aspect of family support in regard to the students’ ability to regulate their emotions and thus imply that family support has great importance in regard to adaptability in the Sino-foreign cooperative education programs. This highlights the importance of not only improving the aspect of emotional support but also emphasizing the aspect of interactive family support.

Looking ahead, in the development of educational and psychological support strategies, it is essential to prioritize enhancing the positive effects of family support on students’ cross-cultural adaptation. However, while family support significantly impacts students’ adaptability, the relationship is indirect, primarily mediated through psychological resilience. Such approaches will assist students in better integrating into complex dual-cultural learning environments and enhance their overall adaptability.

### 5.2 Analysis of the mediating role of psychological resilience

This study finds that psychological resilience plays a crucial mediating role between family support and student adaptability in Sino-foreign cooperative education programs. This mediator role of resilience between external family support and the adaptability of students can influence the students’ reactions and ways of coping with a dual-cultural setting. Model analysis shows that psychological resilience significantly influences student adaptability indirectly. This result suggests that while family support has a direct effect on student adaptability, its impact is substantially enhanced by the internal factor of psychological resilience. These findings are consistent with the research of Zhang et al. (2021), who discovered that psychological resilience can mediate the relationship between external support and student adaptability. Similarly, Qin et al. (2023) support this finding, demonstrating that psychological resilience acts as a full mediator between student mental health and self-efficacy, and enhancing it can boost student adaptability. Psychological resilience is comprised of three dimensions: tenacity, positive cognition, and emotional control, which aid students in handling external pressures and challenges, thereby improving their adaptability in Sino-foreign cooperative education programs. Tenacity enhances students’ perseverance when facing difficulties, while positive cognition helps them maintain an optimistic attitude and manage uncertainties in their academic and personal lives.

Path analysis further confirms the full mediating role of psychological resilience between family support and student adaptability. Essentially, the influence of family support on student adaptability is predominantly transmitted through the mechanism of psychological resilience. This full mediation effect arises because psychological resilience strengthens the impact of family support through enhancing students’ coping mechanisms, emotional regulation, and overall adjustment in cross-cultural settings. This indicates that psychological resilience, as a crucial psychological resource, can transform external support into tangible improvements in adaptability. The mediating role of psychological resilience underscores the importance of not only strengthening family support but also cultivating students’ psychological resilience. Therefore, in Sino-foreign cooperative education programs, interventions such as psychological education and emotional management training can be employed to assist students in better handling environmental changes and challenges, ultimately enhancing their overall adaptability.

### 5.3 Analysis of the internal and external mechanisms affecting student adaptability

This study finds that the adaptability of students in Sino-foreign cooperative education programs is shaped by a combination of internal factors, such as psychological resilience, and external factors like family support. These components interact through intricate mechanisms, creating a multi-dimensional system that influences the development of student adaptability. The family support leads to the psychological resilience of students, which is a key factor in this interaction and an important mediator of the relation between family support and adaptability. Exploring these mechanisms provides deeper insight into how student adaptability evolves.

Firstly, consider the role of internal factors. Psychological resilience serves as a vital internal resource, playing a pivotal role in managing environmental changes and stress. Psychological resilience not only directly impacts student adaptability but also amplifies the effects of external support through mediating processes. Research by Qian and Yu (2023) highlights that psychological resilience acts as a crucial psychological buffer during challenging times, enabling students to maintain a positive outlook and boost their adaptive ability.

Family support, as a crucial external factor, provides significant assurance for the adaptability of students in Sino-foreign cooperative education programs. This study reveals that family support has both direct and indirect effects on students’ adaptability. Specifically, the emotional support and psychological security provided by families are vital in helping students manage the emotional fluctuations and feelings of isolation commonly experienced in these programs. Research by Xian et al. (2023) found that family support can also reduce academic stress and aid students in adapting to bilingual learning environments, significantly improving their overall adaptability. Similarly, Yu et al. (2021) emphasized that family support, by strengthening psychological resilience, can alleviate academic burnout, thereby enhancing students’ adaptability.

This study further explores the interaction between internal and external factors influencing student adaptability. It becomes clear that psychological resilience acts as a mediator that links family support to adaptability, explaining why the direct impact of family support alone is insufficient. It is evident that family support impacts student adaptability in Sino-foreign cooperative education programs both directly and indirectly through the mediating role of psychological resilience. This supports the hypothesis that psychological resilience fully mediates the relationship between family support and student adaptability.

From the analysis, it is clear that the adaptability of students in Sino-foreign cooperative education programs is influenced by a combination of internal and external factors. Within the bicultural context of these programs, a student’s ability to adapt hinges on both external resources, such as family support, and internal psychological resources, such as resilience. To enhance the overall adaptability of students in higher education, it is crucial to strengthen family support and focus on cultivating psychological resilience through psychological interventions and cross-cultural training. This approach promotes the coordinated development of these factors and ultimately improves students’ overall adaptability.

## 6 Conclusions and recommendations

### 6.1 Conclusion

**Figure 4:**
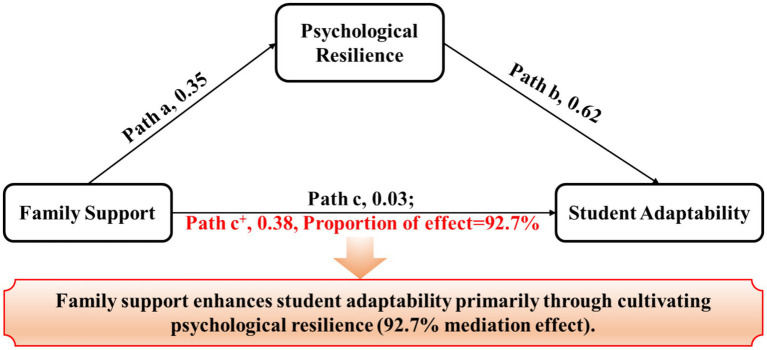
The mediation effect of psychological resilience between family support and student adaptability. Path a, the effect of family support on psychological resilience; Path b, the effect of psychological resilience on student adaptability; Path c, the direct effect of family support on student adaptability; Path c+, the indirect effect of family support on student adaptability when psychological resilience as the mediator.

This study conducted an empirical investigation into the experiences of students from Chengdu’s Sino-foreign cooperative education programs to explore the relationships between family support, psychological resilience, and student adaptability, and to verify the mediating role of psychological resilience between family support and student adaptability. The findings are summarized as follows:

(1) Impact of family support on student adaptability: Emotional support, tangible support, and interactive support from families were all significantly positively correlated with the cultural adaptability, social adaptability, and emotional adaptability of students. Notably, emotional support exerted the most substantial influence on emotional adaptability (*r* = 0.32), highlighting its crucial role in enhancing students’ emotional regulation capabilities.
(2) As shown in the Figure 4 and Table 4, Mediating Role of Psychological Resilience: Psychological resilience, through its dimensions of tenacity, positive cognition, and emotional control, significantly influenced all dimensions of student adaptability. Emotional control showed the greatest impact on emotional adaptability (*r* = 0.45). Psychological resilience fully mediated the relationship between family support and student adaptability, with an indirect predictive effect of 0.38, accounting for 92.7% of the total effect.
(3) Structural equation modeling analysis: The analysis indicated that the direct impact of family support on student adaptability was relatively minor. However, through the mediating role of psychological resilience, its indirect impact on student adaptability was significant. This further validates the mediating role of psychological resilience in the relationship between external support and student adaptability.

**Table 4:** Bootstrap analysis of mediating effect of psychological resilience between family support and student adaptability.

Effect categories	Effect path	*β*	Proportion of effect (%)
Total effect	—	0.41	—
Direct effect	Family support → student adaptability	0.03	7.3%
Indirect effect	Family support → psychological resilience → student adaptability	0.38	92.7%

### 6.2 Recommendations

Based on these findings, several recommendations can be made to enhance the adaptability of students in Sino-foreign cooperative education programs:

(1) Strengthen family support

Involvement of the family in the development of adaptability among students is very important. In order to develop this support in the context of Sino-foreign cooperation in education, the university should cooperate with the family to deliver specific emotional support. For instance, the universities should organize orientations and workshops for the families. These can involve educating the parents on the cultural and academic challenges the children will encounter when exposed to the cultural environment of the cross-cultural setting. Moreover, the universities should develop programs related to involvement between the family and the peer group. This will enable the students and their families to get involved in activities that aim to build resilience among the students.

(2) Promote psychological resilience

Institutions should include psychological resilience training programs that cater specifically to the requirements of the students in the Sino-foreign cooperative education system. Workshops can be conducted for the students pertaining to the techniques of managing their emotions and changing the way their minds react to a given situation. Psychological resilience training can be conducted during the orientation program that focuses specifically on adaptability in cross-cultural societies and stress management as a student in the aforementioned programs. In addition to that, students’ resilience peer support groups should be created where the students can share their experiences of how they manage the situation and at the same time can emotionally support each other. This will play a pivotal role in dealing with the challenges faced as students in bi-cultural and bi-lingual programs.

(3) Integrated support systems

Sino-foreign cooperative education programs should create a more holistic support network through the integration of psychological counseling services, academic advising services, and cultural adaptation services. Universities can contract cultural organizations to offer the students cultural immersion experiences that can offer them the navigation keys to the cultural ways of the partnering countries. Tailor-made academic support structures should subsequently be implemented to factor in the peculiar circumstances under which the students find themselves because of the adjustment of their educational expectations from their home countries as well as the partnering countries. Creating mentorship programs involving senior students from the same cultural backgrounds as the new entrants can also offer important support services, particularly related to the management of their emotions and adaptation to academics.

(4) Continuous monitoring and feedback

For the efficiency of the support structures that have been put in place, monitoring the adaptability and resilience of the students became important. This can be achieved through the conducting of quarterly surveys among the students to evaluate their adaptability and resilience. In order to monitor their concerns and initiate corrective measures, the establishment of real-time feedback systems like focus group meetings or personalized counseling meetings became important. For the evaluation of the efficiency of the support structures annually put in place, review meetings based on the input gained from the students became important.

By implementing these recommendations, educational institutions can better support the adaptability of students in Sino-foreign cooperative education programs, ultimately enhancing their academic success and overall wellbeing.

### 6.3 Countermeasures and suggestions

To enhance the adaptability of students in Chengdu’s Sino-foreign cooperative education programs, this article proposes the following strategies:

(1) Building a family-centered support system

In implementing Sino-foreign cooperative education programs, universities should actively foster collaboration between families and educational institutions and systematically develop a family-centered support system. Emphasizing emotional support as the primary focus, the positive role of parents in facilitating students’ cross-cultural adaptation should be accentuated. Universities could regularly organize family education training sessions and home-school communication meetings to equip parents with effective emotional support techniques, thereby bolstering students’ psychological security when facing academic challenges and cultural differences. Additionally, concerning tangible support within the family support framework, schools should address the concerns of economically disadvantaged students by establishing special funding initiatives or providing living allowances, ensuring that students receive adequate material support in Sino-foreign cooperative education programs. Furthermore, the continuity and effectiveness of interactive support are crucial. To enhance this, universities can utilize various online and offline channels to establish robust home-school communication platforms. By conducting regular parent open days and online consultations, a smooth flow of information can be maintained between parents, teachers, and students, thereby forming an effective support network within the family and enhancing students’ overall adaptability.

(2) Comprehensive promotion of psychological resilience education

Psychological resilience is an essential psychological resource for students to manage the pressures and challenges inherent in the cross-cultural environment of Sino-foreign cooperative education programs. Educational institutions should integrate psychological resilience education into their comprehensive teaching management systems, offering systematic training and guidance. Initially, schools can introduce mental health courses and emotional management workshops to enhance students’ resilience in facing academic and life pressures. This support also aids them in maintaining focus within a bilingual teaching context. Furthermore, colleges and universities should actively implement cognitive behavioral therapy and psychological counseling sessions tailored to the nuances of psychological education, guiding students to develop positive cognitive patterns and bolster their confidence and psychological flexibility in navigating the challenges of cooperative education programs. Additionally, institutions need to regularly provide specialized emotional management training to strengthen students’ emotional regulation capabilities, enabling them to more effectively tackle the challenges presented in Sino-foreign cooperative education environments and improve their overall adaptability.

(3) Optimization of the management mechanism for Sino-foreign cooperative education programs

At the educational management level, colleges and universities should refine the overall management mechanisms of Sino-foreign cooperative education programs to systematically enhance students’ adaptability. Firstly, institutions should establish a tracking and assessment mechanism to promptly gauge students’ adaptability, regularly evaluating students’ adaptation status within the cooperative education program and modifying teaching strategies and support measures based on the assessment results. Secondly, colleges should intensify specialized training for teachers to augment their sensitivity and support capabilities, ensuring that the teaching staff can effectively recognize and address various issues students encounter in their learning journey. Moreover, institutions should bolster personalized support for students in daily management by offering a range of cultural adaptation activities tailored to individual student needs. This approach helps students better integrate into the bilingual learning environment and promotes their holistic development.

## Data Availability

The original contributions presented in the study are included in the article/supplementary material, further inquiries can be directed to the corresponding author.
